# Angiotensin II increases respiratory rhythmic activity in the preBötzinger complex without inducing astroglial calcium signaling

**DOI:** 10.3389/fncel.2023.1111263

**Published:** 2023-02-02

**Authors:** Charlotte Tacke, Anne M. Bischoff, Ali Harb, Behnam Vafadari, Swen Hülsmann

**Affiliations:** Department of Anesthesiology, University Medical Center, Georg-August University, Göttingen, Germany

**Keywords:** preBötzinger complex (preBötC), angiotensin II, respiration, respiratory activity, astrocytes

## Abstract

Angiotensin II (Ang II) is the primary modulator of the renin-angiotensin system and has been widely studied for its effect on the cardiovascular system. While a few studies have also indicated an involvement of Ang II in the regulation of breathing, very little is known in this regard and its effect on brainstem respiratory regions such as the preBötzinger complex (preBötC), the kernel for inspiratory rhythm generation, has not been investigated yet. This study reports that Ang II temporarily increases phrenic nerve activity in the working heart-brainstem preparation, indicating higher central respiratory drive. Previous studies have shown that the carotid body is involved in mediating this effect and we revealed that the preBötC also plays a part, using acute slices of the brainstem. It appears that Ang II is increasing the respiratory drive in an AT1R-dependent manner by optimizing the interaction of inhibitory and excitatory neurons of the preBötC. Thus, Ang II-mediated effects on the preBötC are potentially involved in dysregulating breathing in patients with acute lung injury.

## 1. Introduction

Angiotensin II (Ang II), the main modulator of the renin-angiotensin system (RAS), has a multitude of biological roles but is probably best known for its effect on the cardiovascular system, especially blood pressure regulation. In the current COVID-19 pandemic, the RAS has gained further attention as the angiotensin-converting enzyme 2 (ACE2) is the functional receptor for the viral target cell entry (Hoffmann et al., 2020; Zamorano Cuervo and Grandvaux, 2020). Moreover, dysregulation of RAS, e.g., increased Ang II levels, has been associated with acute lung injury and the pathogenesis of acute respiratory distress syndrome (ARDS) (Imai et al., 2005; Wösten-van Asperen et al., 2011; Zou et al., 2014). Studies have also indicated a role of the RAS in the regulation of breathing. Ang II has been shown to stimulate the afferent activity of the carotid body (CB), playing an important role in the control of breathing and in the autonomic control of cardiovascular functions (Allen, 1998; Schultz, 2011). Furthermore, peripheral infusion of Ang II in dogs stimulates respiration (Ohtake et al., 1993; Ohtake and Jennings, 1993), which is however, mediated only in part by the CB (Potter and McCloskey, 1979), suggesting a potential involvement of respiratory control regions in the brainstem. Ang II can interact with the brain *via* the circumventricular organs (CVO) (Mendelsohn et al., 1988; Fry and Ferguson, 2007), which lack a blood-brain barrier. The CVO projects to the rostral ventrolateral medulla (RVLM), a region having a key role in the regulation of blood pressure and respiration. All components of the RAS can also be locally produced within the brain (Ganten and Speck, 1978; Allen et al., 1998; McKinley et al., 2003; Li et al., 2012; Wright and Harding, 2013; Shinohara et al., 2016). Injection of Ang II into the nucleus of the solitary tract, which relays CB input to other brainstem respiratory control regions, was shown to increase the respiratory rate in rats (Paton and Kasparov, 1999). Furthermore, Ang II signaling *via* its main receptor, the angiotensin II type 1 receptor (AT1R), increases the firing rate of neurons in brain areas involved in controlling the cardiovascular system, including the subfornical organ (SFO), paraventricular nucleus of the hypothalamus (PVN), organum vasculosum of the lamina terminalis (OVLT) (Felix and Schlegel, 1978; Suga et al., 1979; Knowles and Phillips, 1980; Harding and Felix, 1987; Cancelliere and Ferguson, 2017) and the RVLM (Chan et al., 1991; Matsuura et al., 2002; Dampney et al., 2007). The PVN and SFO also regulate respiration (Ferguson et al., 1989; Yeh et al., 1997). While the modulatory effect of Ang II on cardiovascular regions of the brain has been widely studied (Baltatu et al., 2000), its effect on neurons of key respiratory regions, such as the preBötzinger complex (preBötC) has not been investigated yet.

In this study we aim to understand the role of Ang II in regulating the neuronal activity in the core of the respiratory network, the preBötC, responsible for respiratory rhythm generation and control of inspiration. We show for the first time, that Ang II signaling *via* the AT1 receptor increased the rhythmic activity in the preBötC in acute slices of the brainstem. Furthermore, activation of the AT2 and Mas receptors, typically described to counteract the functions of the AT1R, did not affect rhythmic activity. On the cellular level, we show that only the activity of neurons, but not astrocytes, is modulated by Ang II and that the effects elicited by Ang II involve direct actions on inhibitory and excitatory neurons.

## 2. Materials and methods

### 2.1. Animals

This study was carried out in accordance with the guidelines for the welfare of experimental animals issued by the European Communities Council Directive (2010/63/EU) and with the German Protection of Animals Act (Tierschutzgesetz; TierSchG). All the procedures were approved by the Animal Welfare Commission of the University Medical Center Göttingen, Germany and the Niedersächsische Landesamt für Verbraucherschutz und Lebensmittelsicherheit (LAVES). All mice used in this study were bred in the central animal facility of the University Medical Center Göttingen.

For the identification of glycinergic neurons by the expression of EGFP under the control of the GlyT2 promoter and GABAergic neurons by the expression of tdTomato under the GAD65 promoter we used the COFLUOR line Tg(Gad2-tdTomato)DJhi × Tg(Slc6a5-EGFP)1Uze (Mesuret et al., 2018; Hirrlinger et al., 2019). For identification of astrocytes two different mouse lines were used. One of them were TgN(GFAP-EGFP)GFEC-FKi mice, expressing EGFP under the control of the human GFAP promotor (Nolte et al., 2001; Grass et al., 2004). The other line used for identifying astrocytes were Aldh1l1-GCaMP6s mice [Tg(Aldhll1-cre/ERT2)02Kan x B6; 129S-B6J.Cg-Gt(*ROSA)26Sor^TM^*^96(CAG–GCaMP6*s)He*^/MwarJ], expressing GCaMP6s conditionally in astrocytes (Madisen et al., 2015; Winchenbach et al., 2016). B6; 129S-B6J.Cg-Gt(*ROSA)26Sor^TM^*^96(CAG–GCaMP6*s)He*^/MwarJ were purchased from Jaxon laboratory (JAX stock #024106).

For the initial pharmacological analysis of the effect of Ang II (including the inhibition or activation of different receptors), mice form different lines were used. These lines included wild-type C57Bl/6J mice but also transgenic lines without potential harmful phenotype, that are used in our lab in other projects. To minimize a wastage of animals with unfavorable genotype (3R), we used mice from the modified COTRIND line [TgN(Gad65-NCreERT2) × TgN(GlyT2-ERT2CCre) × 129S6-Gt (ROSA) × 26Sortm9(CAG-tdTomato)Hze/J × B6;129S-Gt(ROSA) 26Sortm32.1(CAG-COP4*H134R/EYFP)Hze/J] (Hirrlinger et al., 2019) and/or mice from the Aldh1l1-GCaMP6s line, which had not been exposed to tamoxifen.

### 2.2. Working heart-brainstem preparation

The working heart-brainstem preparation (WHBP) was performed as previously described (Mesuret et al., 2018). Briefly, adult mice (over 3 months old) of either sex received an overdose of isoflurane. After confirmation of apnea and the absence of the nociceptive withdrawal reflex, they were then bisected below the diaphragm for exsanguination. Furthermore, the cerebellum was removed and a decerebration at the parafollicular level was performed. The descending aorta and thoracic phrenic nerve (PN) were isolated and cut distally. The preparation was placed in a recording chamber and the descending aorta was cannulated and retrogradely perfused at a flow rate of 16–20 ml/min using a peristaltic pump (Watson Marlow) with carbogenated (95% O_2_, 5% CO_2_) artificial cerebrospinal fluid (ACSF). The composition of the ACSF was (in mM): 125 NaCl; 25 NaHCO_3_; 2.5 CaCl_2_; 1.25 MgSO_4_; 1.25 KH_2_PO_4_; 5 KCl; 10 glucose, pH 7.4 at 32°C. For the optimization of the osmotic pressure the oncotic agent Ficoll (1.25%; Sigma–Aldrich) was supplemented. The perfusion pressure was monitored using the second lumen of the catheter. At the beginning of the experiment, the flow of the perfusate was adjusted to observe uniform phrenic nerve discharges, thereafter, no further adjustment was allowed. Phrenic nerve activity (PNA) was recorded using custom-made borosilicate glass suction electrodes. PNA signals were amplified 12,500 times and band-pass filtered (0.25–2 kHz) using a custom-made amplifier (electronic workshop of the department of physiology, Göttingen). Signals were digitized by a PowerLab 8/30.

### 2.3. Rhythmic slice preparation

Rhythmic slices were prepared from mice between postnatal day 4–12 (P4–P12) of either sex as previously described (Hülsmann et al., 2000; Winter et al., 2009). Briefly, mice under isoflurane anesthesia were rapidly decapitated and the brainstem was isolated in ice-cold carbogenated (95% O_2_, 5% CO_2_) ACSF (in mM: 118 NaCl, 3 KCl, 1.5 CaCl_2_, 1 MgCl_2_, 1 NaH_2_PO_4_, 25 NaHCO_3_, and 30 D-Glucose; pH 7.4). The dorsal surface of the brainstem with the rostral end facing up, was fixed to an agar block, cut at a ∼15° angle, with cyanoacrylate glue (Loctite, Germany). Transverse slices containing the preBötC on the rostral side were sectioned with a vibratome (Leica VT 1200S, Leica Biosystems, Nussloch, Germany) at a thickness of 600 μm in ice-cold carbogenated ACSF. After a recovery period of at least 30 min in a holding chamber at room temperature (RT), the slice was transferred to a submerged recording chamber mounted on an upright microscope (Axioscope FS, Zeiss, Germany) and was again superfused with carbogenated ACSF at 30°C. The slice was fixed with a custom-made nylon fiber grid. Rhythmic activity was induced and maintained by elevating the potassium concentration to 8 mM (Smith et al., 1991).

### 2.4. Electrophysiological recordings

The respiratory rhythmic activity was recorded extracellular with microelectrodes filled with ACSF. The recording electrodes were pulled from borosilicate glass capillary (0.86 mm × 1.50 mm × 100 mm, Science Products GmbH) with a DMZ Universal Electrode Puller (Zeitz-Instruments, Munich, Germany). Local field potentials (LFP) were amplified by a custom-build amplifier (5000–20000 times) band-pass filtered (0.25–3.5 kHz) and digitized at 10 kHz on Digidata interface using pClamp10 software (Molecular Devices Inc., RRID:SCR_011323). For parallel calcium imaging experiments the trigger pulse for each image frame was recorded simultaneously with the LFP signal for later offline analysis.

### 2.5. Cell loading for calcium imaging

Multi-cell bolus loading of the preBötC was performed with the calcium dye Oregon Green 488 BAPTA-1 AM (OGB, Thermo Fisher Scientific, Cat# O6807). A total of 50 μg OGB were dissolved in 48 μl DMSO (Sigma-Aldrich, Cat# D5879) and stored at −20°C in 4 μl aliquots until usage. One aliquot was mixed with an equal volume of DMSO containing 10% Pluronic F-127 stored at RT (Thermo Fisher Scientific, Cat# P6867) and 8.6 μl of an extracellular solution (in mM: 150 NaCl, 2.5 KCl and 10 HEPES; pH 7.4). The OGB solution, with a final concentration of 414 μM, was pressure injected (0.2 bar) below the slice surface into the preBötC for 10 min, followed by an incubation period of 30 min for sufficient dye loading into the cells and to allow washout of excess extracellular dye.

### 2.6. Calcium imaging using 2-photon microscopy

Imaging was performed with a 2-photon (2P) laser-scanning microscope (TriMScope, LaVision, BioTec, Bielefeld, Germany) using a 20× (1.0 NA) water immersion objective lens (Zeiss, Oberkochen, Germany) and GaAsP photomultipliers for non-descanned detector (Hamamatsu Photonics K.K., Hamamatsu, Japan). Two photon excitation was achieved with a Ti:Sapphire Laser (MaiTai BB, SpectraPhysics, Santa Clara CA, USA). Before and after calcium imaging, reference images were taken for COFLOUR (tdTomato, EGFP, and OGB) and hGFAP-EGFP (EGFP and OGB) mice. For excitation three wavelengths were used: 720, 800, or 900 nm and emitted fluorescence was simultaneously detected through three kinds of band-pass emission filters: 641/75, 531/40, or 475/50 nm. Spectra overlapping of fluorescence was decomposed off-line (see section “2.7. Image processing”). For calcium imaging, OGB fluorescence was detected through a 531/40 nm band-pass emission filter with excitation at 800 nm wavelength or 915 nm for GCaMP6s (Aldh1l1-GCaMP6s mice). Calcium imaging recordings were performed at 6–8 Hz. Optical filters were obtained from AHF Analysentechnik AG (Tübingen, Germany). All settings were controlled by Imspector Software (RRID:SCR_015249).

### 2.7. Image processing

All “Imspector”-images were exported to TIFF format and analyzed using ImageJ (RRID:SCR_003070) and MATLAB (RRID:SCR_001622). Considering spectral overlapping of fluorescence from tdTomato, EGFP and OGB (COFLOUR mice), we used a spectral unmixing plug-in for ImageJ to separate the signal from the three fluorophores using non-negative tensor factorization (Neher et al., 2009). A custom-written MATLAB script was used to perform drift-correction of the calcium recordings and for generating cross-correlation maps of the LFP and calcium responses. Therefore, the maximum normalized cross-correlation coefficient (maxCC) between bursting pattern of integrated LFP and fluctuation of OGB fluorescence at each pixel was calculated (Oke et al., 2018). In order to improve signal to noise ratio, the images were filtered spatially by taking the unweighted average of a 2 × 2 region around each pixel using the “Median 3D” filter in ImageJ.

### 2.8. Analysis of LFP and PNA recordings

The LFP and PNA were measured over time and rectified, integrated (time constant decay 150 and 250 ms, respectively) and analyzed using LabChart8 (ADInstruments). For frequency analysis the number of bursts was counted. For graphs showing frequency and amplitude over time data after application of the treatment was normalized to the baseline (control condition).

### 2.9. Analysis of calcium recordings

Somatic calcium changes were analyzed using the “multi measure” regions of interest (ROI)-macro from ImageJ. ROI-intensities of individual cell somata were expressed as relative changes (ΔF/F0), for which F0 was defined as the first 20 s after start of the recording without calcium activity (otherwise the time frame was shifted to a period during control without calcium activity). A cell was determined to have responded to the treatment, if the mean during the treatment (average of 30–60 s) was greater than 2× the standard deviation (SD) of the mean from the baseline (control condition; 30 s).

### 2.10. Tamoxifen injection

For calcium imaging experiments in astrocytes using Aldh1l1-GCaMP6s mice, lactating dams were injected intraperitoneally with Tamoxifen (1 mg/100 μl, dissolved in corn oil; Sigma–Aldrich Cat# T5648-1G, Cas# 10540-29-1) on two consecutive days, administering tamoxifen to pups through the milk of the lactating dams, beginning when the pups were one day old (P1).

### 2.11. Pharmacology

Angiotensin II (Ang II; Tocris Cat# 1158), Ang-(1–7) (Tocris Cat# 1562), AT2R-Agonist CGP42112 (Tocris Cat# 2569), Losartan potassium (Tocris Cat# 3798) and (+-)-Norepinephrine (+)-bitartrate salt (Sigma Cat# A0937, Cas # 3414-63-9) were dissolved in sterile H_2_O. Ang II and CGP 42112 were used at a final working concentration of 500 nM, Ang-(1–7) at 10 nM, losartan at 5 μM and norepinephrine at 10 μM.

Regarding all pharmacological LFP experiments with Ang II, Ang-(1–7), AT2R-Agonist and losartan, rhythmic activity was recorded for 15 min under control condition. Treatment was applied for 20 min followed by a 40 min washout (if performed), with one exception. In one experiment losartan was applied for 10 min after which Ang II was co-applied for another 20 min. Regarding the calcium imaging experiments, calcium activity was recorded under control condition for 30–60 s after which Ang II was applied and recorded for another ∼7 min. Immediately after this, a new recording was started in which the calcium activity under the previously applied Ang II was recorded again for 30–60 s, then norepinephrine was co-applied.

### 2.12. Statistical analysis

**FIGURE 1:**
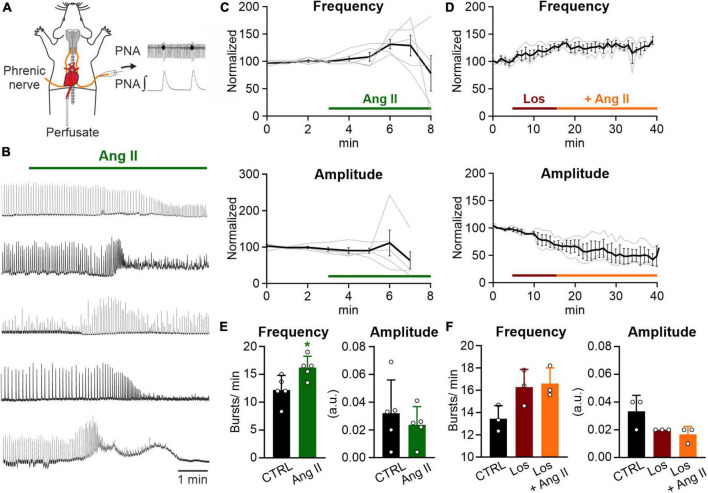
Ang II increases respiratory rate in the WHBP. **(A)** Illustration of the working heart-brainstem preparation (WHBP) showing recordings of phrenic nerve activity (PNA). **(B)** Recordings of the integrated PNA in response to Angiotensin II (Ang II) treatment. **(C,D)** Graphs show the frequency and amplitude of PNA over time. Signals after treatment were normalized to the baseline (control condition). Gray traces show each individual recording and the black trace the mean value. **(C)** Treatment with Ang II alone (100–500 nM; *N* = 5 mice, 3 male and 2 female) and **(D)** a 12 min pre-treatment with losartan (Los; 5 μM) followed by the co-application of Ang II (500 nM; *N* = 3 female mice). **(E)** Charts show the averaged data of 2 min time intervals for the different conditions for frequency and amplitude (CTRL 1–3 min, Ang II 6–8 min). **(F)** Charts show the averaged data of a 3 min time interval for CTRL and a 5 min time interval for the other two conditions (CTRL 2–5 min, Los 12–17 min, and Ang II 21–26 min). The data are presented as mean ± SD and were tested using a **(E)** paired Student’s *t*-test or **(F)** Friedman test. *p < 0.05.

Statistical analysis and graph plotting were preformed using GraphPad Prism 9 (RRID:SCR_002798). Data are presented as mean ± standard deviation (SD). *N* = number of animals/slices. Data were tested for normal distribution (Anderson–Darling test, D’Agostino-Pearson test, Kolmogorov–Smirnov test, and Shapiro–Wilk test). For comparisons of two groups, a paired Student’s *t*-test was used. For the comparison of more than two groups a repeated measures (RM) one-way ANOVA followed by a Bonferroni’s multiple comparison *post hoc* test was used. The only exception is Figure 1F, for which a non-parametric test was performed: Friedman test followed by a Dunn’s multiple comparison test. Data were considered statistically significant with *p* < 0.5.

## 3. Results

### 3.1. Angiotensin II increases the respiratory rate in the intact respiratory network

It has been shown that peripheral infusion of Ang II stimulates respiration in dogs (Ohtake et al., 1993; Ohtake and Jennings, 1993) and that Ang II stimulates the afferent activity of the CB, playing an important role in the control of breathing (Allen, 1998; Schultz, 2011). Therefore, to assess the effect of Ang II on respiratory network activity, we recorded phrenic nerve activity (PNA) in an arterially perfused working heart-brainstem preparation (WHBP; Figure 1A). Ang II (100–500 nM) temporally increased the frequency of PNA significantly (Figure 1E; CTRL: 12.2 ± 2.5 min^–1^ vs. Ang II: 16.2 ± 2.0 min^–1^, *p* = 0.022). The response peaked 3–4 min after the application of Ang II (Figure 1C). Interestingly, this response of the network was not persistent and a decline of the amplitude occurred, which hampered the detection of the bursts (Figure 1B). Pre-application of the AT1R antagonist losartan for 10 min abolished the Ang II mediated increase in frequency of PNA (Figures 1D, F; Friedman test *p* = 0.194, Dunn’s adjusted *p*-value: *p* = 0.083).

The increase in PNA activity indicates higher central respiratory drive, leaving open the question whether the effect of Ang II is mediated solely by the CB or if respiratory control centers in the brainstem are involved as well. We also cannot exclude, that the systemic application of Ang II, which is a potent vasoactive agent (Vincent et al., 2005; Noureddine et al., 2020), might cause vasoconstriction, thereby reducing perfusion and oxygenation in the brainstem. Indeed, as shown in the traces in Figure 1B the initial increase in phrenic burst frequency is followed by some degree of depression – a response pattern commonly observed in studies exposing the *in situ* preparations to long periods of hypoxia. Therefore, to exclude that the observed effect is a consequence of brainstem ischemia we continued our study in acutely isolated brain slices.

### 3.2. Ang II increases rhythmic activity in the preBötC

**FIGURE 2:**
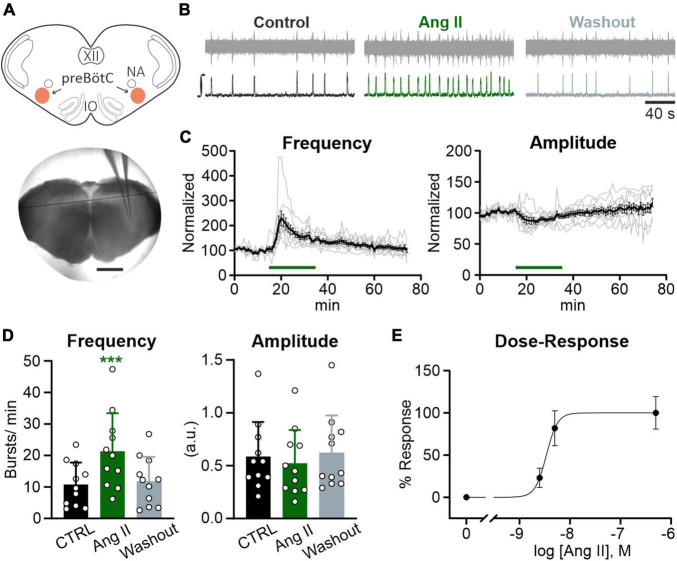
Ang II modulates rhythmic activity in the preBötC. Local field potential (LFP) recordings were performed in the preBötC in rhythmic acute brainstem slices (600 μm) from mice (P4–P12). **(A)** Schematic drawing of a brainstem slice with the preBötC marked in red (NA: nucleus ambiguus, IO: inferior olive) and an acute slice with the recording electrode placed in the preBötC. Scale bar 1 mm. **(B)** Example traces of the recorded (upper traces) and integrated (lower traces) LFP for each condition. Basic rhythmic activity was recorded for 15 min (control), followed by a 20 min Ang II treatment and a 40 min washout. **(C)** Recordings over time. Data after application of Ang II (500 nM) was normalized to the baseline (control condition). Gray traces show each individual recording (*N* = 10 slices) and the black trace the mean value. **(D)** Charts show the averaged data of 5 min intervals for the different conditions (CTRL 10–15 min, Ang II 20–25 min, Washout 70–75 min). The data are presented as mean ± SD and were tested using a RM one-way ANOVA. ***p < 0.001. **(E)** Dose-Response graph of 2.5 nM (*N* = 7 slices), 5 nM (*N* = 6 slices), and 500 nM Ang II. The frequency was used for this evaluation.

In the next set of experiments, we addressed the question whether the Ang II effect is mediated *via* modulation of neuronal activity in the core of the respiratory network, the preBötzinger complex (preBötC). To assess the effect of Ang II on the rhythmic activity in the preBötC, recordings of the local field potential (LFP) in rhythmic acute brainstem slices (Figure 2A), which retain sufficient circuitry from the respiratory medulla to spontaneously generate inspiratory rhythmic bursts, were performed (Smith et al., 1991; Feldman and Del Negro, 2006). Ang II (500 nM) was applied for 20 min after recording the LFP for 15 min under control conditions, followed by a washout for 40 min. As shown in the LFP traces (Figure 2B), Ang II treatment increased rhythmic activity, reaching the peak 5 min after the start of Ang II application (Figure 2C). The change in frequency was significant compared to the control (Figure 2D; 10.8 ± 7.0 min^–1^ vs. 21.3 ± 12.1 min^–1^, RM one-way ANOVA, Bonferroni adjusted *p*-value: *p* = 0.0003). The initial drop of burst amplitude after Ang II application (Figure 2C, right panel) did not reach significance (Figure 2D; Bonferroni adjusted *p*-value: *p* = 0.065). At the end of the washout, burst frequency returned to control levels (Figure 2D; CTRL: 10.8 ± 7.0 min^–1^ vs. Washout: 11.8 ± 7.7 min^–1^, Bonferroni adjusted *p*-value: *p* = 0.675).

Lower concentrations of Ang II also led to an increase in frequency (Figure 2E), which was significant for a 100× lower concentration of Ang II (5 nM) (6.1 ± 4.7 min^–1^ vs. 10.9 ± 5.4 min^–1^, Student’s paired *t*-test, *p* = 0.009, *N* = 5 slices) but not for a 200× lower concentration (2.5 nM) (4.7 ± 1.0 min^–1^ vs. 5.8 ± 0.6 min^–1^, Student’s paired *t*-test, *p* = 0.084, *N* = 7 slices).

### 3.3. The actions of Ang II are mediated by AT1R activation

**FIGURE 3:**
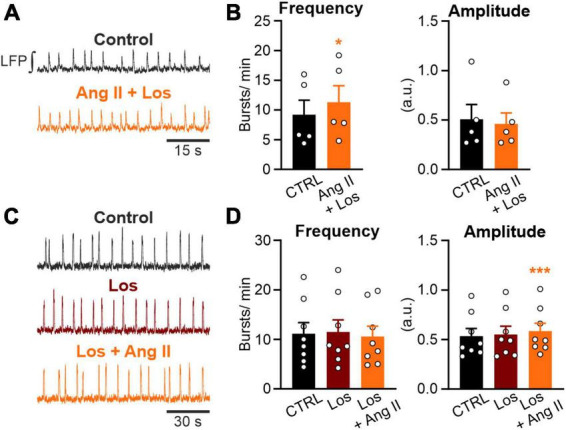
Ang II increases rhythmic activity in an AT1R-dependent manner. LFP recordings in the preBötC in rhythmic acute brainstem slices. **(A,C)** Example traces of the integrated LFP for each condition. Control condition was recorded for 15 min followed by a 20 min treatment, except **(C)** where losartan (Los; 5 μM) was pre-applied for 10 min. **(B,D)** Charts show the averaged data of 5 min intervals. CTRL 10–15 min, **(B)** Ang II + Los 20–25 min, **(D)** Los 20–25 min, Los + Ang II 30–35 min. **(B)** *N* = 5 slices and **(D)** *N* = 8 slices. The data are presented as mean ± SD and were tested using a **(B)** paired Student’s *t*-test or **(D)** a RM one-way ANOVA. *p < 0.05; ***p < 0.001.

Next, we investigated if the AT1R, mediating most of the physiological actions of Ang II, is necessary for the Ang II induced increased rhythmic activity of neurons in the preBötC. Co-treatment of Ang II and the AT1R antagonist losartan (5 μM) strongly reduced the effect of Ang II (Figures 3A, B). While Ang II treatment alone led to an increase in frequency of 97% (Figure 2D), treatment together with losartan reduced this effect to 23%. However, rhythmic activity was still significantly increased (Figure 3B; 9.2 ± 5.4 min^–1^ vs. 11.3 ± 6.2 min^–1^, *p* = 0.011). A 10 min pre-treatment with losartan prevented the increase in frequency induced by Ang II (Figures 3C, D). However, there was a very small (Figure 3D; 7.8 ± 9.6%) but significant increase of the burst amplitude (Figure 3D; Los: 0.55 ± 0.24 vs. Los + Ang II: 0.58 ± 0.23, RM one-way ANOVA, Bonferroni adjusted *p*-value: *p* = 0.001), suggesting some minor AT1R-independent effect of Ang II.

**FIGURE 4:**
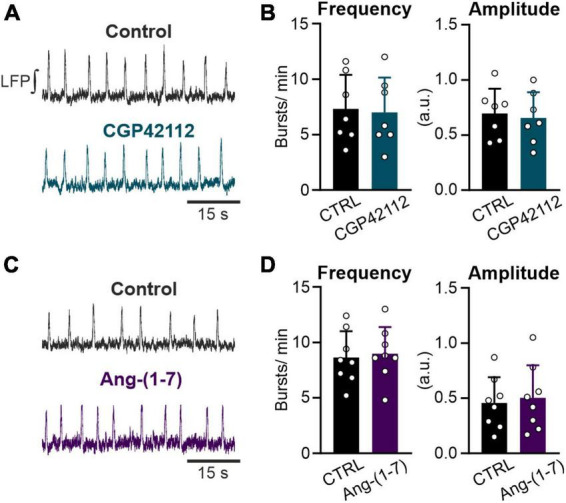
Both the AT2R and the MasR do not modulate respiratory rate. LFP recordings in the preBötC in rhythmic acute brainstem slices. **(A,C)** Example traces of the integrated LFP for each condition. Control condition was recorded for 15 min followed by a 20 min treatment with **(A,B)** the AT2R agonist CGP42112 (500 nM) and **(C,D)** the MasR agonist Ang-(1–7) (10 nM). **(B,D)** Charts show the averaged data of 5 min intervals (CTRL 10–15 min and treatment 30–35 min). **(B)** *N* = 7 slices and **(D)** *N* = 8 slices. The data are presented as mean ± SD and were tested using a paired Student’s *t*-test.

Activation of the AT2R, another functional receptor for Ang II, and the Mas receptor (MasR), binding the Ang II conversion product Ang-(1–7), have been described as eliciting the opposite functions to the AT1R. Therefore, we investigated if activation of these two receptors modulates rhythmic activity in neurons of the preBötC as well. However, neither the activation of the AT2R (500 nM; Figures 4A, B) nor the MasR (10 nM; Figures 4C, D) had any significant influence on the frequency of rhythmic bursts.

Taken together, the results show that the Ang II elicited stimulation of the network activity is mediated by AT1R activation.

### 3.4. No recruitment of additional rhythmic neurons by Ang II

**FIGURE 5:**
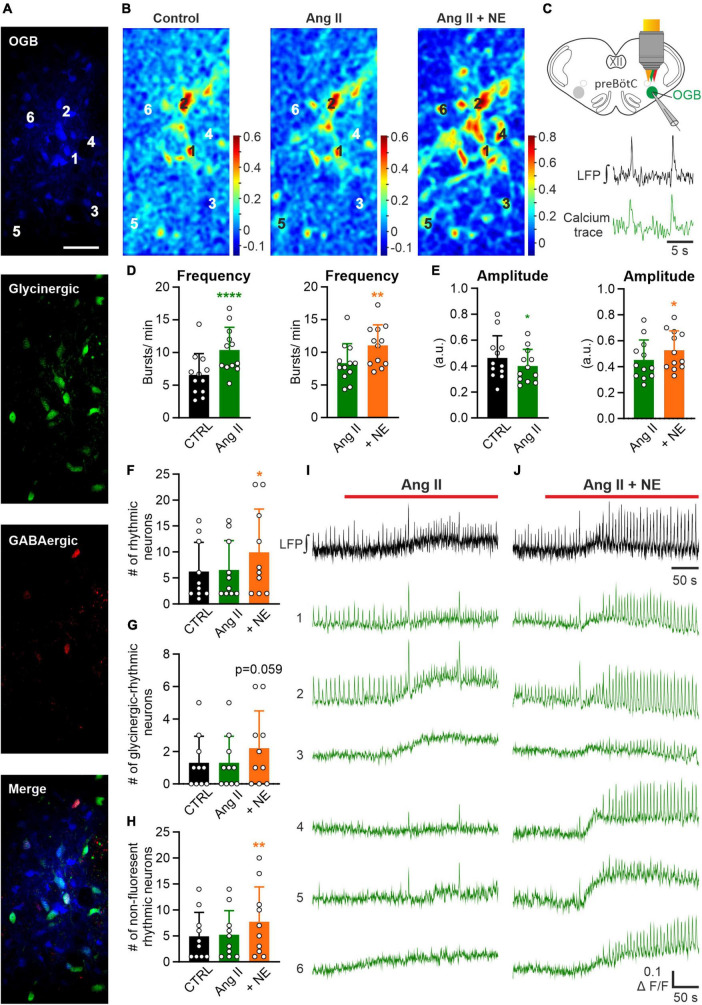
Effect of Ang II is not mediated by recruitment of additional rhythmic neurons. LFP recordings were combined with 2P microscopy in acute rhythmic slices. Oregon Green 488 BAPTA-1AM (OGB) was pressure injected into the preBötC. **(A)** Images of neurons loaded with OGB from COFLOUR mice expressing EGFP in glycinergic and tdTomato in GABAergic interneurons. Scale bar 50 μm. **(B)** Cross-correlation maps of the LFP and calcium responses for the different treatment groups. Norepinephrine (NE; 10 μM) was added to Ang II. **(C)** Schematic drawing of an OGB injected slice, showing LFP and calcium traces of a rhythmic neuron below. **(D,E)** The baseline of the LFP was recorded for 5 min before the treatment was applied. Charts show averaged LFP data from imaging experiments (control condition was recorded for 3 min–time interval 2–5 min and the treatment groups for 4 min–time interval 8–12 min), *N* = 12 slices. **(F)** Chart shows the mean number of all rhythmic neurons, which is then divided into **(G)** glycinergic-rhythmic and **(H)** non-fluorescent rhythmic neurons. *N* = 10 slices. The data are presented as mean ± SD and were tested using a **(D,E)** paired Student’s *t*-test or a **(F–H)** RM one-way ANOVA. *p < 0.05; **p < 0.01; ****p < 0.0001. **(I,J)** LFP (black) and calcium traces (green) from selected neurons **(A)** treated with Ang II and Ang II + NE.

In the next set of experiments, we investigated the effect of Ang II on the cellular level in the preBötC. Therefore, LFP recordings were combined with 2P microscopy calcium imaging experiments in rhythmic acute slices pressure injected with Oregon Green 488 BAPTA-1AM (OGB) into the preBötC (Figure 5C) using double-transgenic mice (COFLOUR) expressing EGFP in glycinergic and tdTomato in GABAergic interneurons (Figure 5A). Calcium activity and LFP were recorded under control condition, after which Ang II was applied, followed by the co-application of norepinephrine (NE, 10 μM), used as a positive control. The co-application of norepinephrine significantly increased burst frequency (Figure 5D, *p* = 0.004) and amplitude (Figure 5E, *p* = 0.025) of the LFP compared to Ang II. Cross-correlation maps (Figure 5B) between the LFP and the calcium recordings show that the co-application of norepinephrine led to a visible increase in the number of cells with rhythmic activity, while no difference was visible with Ang II treatment alone (Figure 5B). The exemplary calcium traces (Figures 5I, J) show neurons with rhythmic activity already under control condition (traces 1–2) and neurons, in which rhythmic activity was induced (traces 3–6) with the co-application of norepinephrine. Indeed, norepinephrine significantly increased the number of additional recruited rhythmic neurons compared to the control (Figure 5F; RM one-way ANOVA, Bonferroni adjusted *p*-value: *p* = 0.009) while Ang II did not. In total 62 neurons were rhythmically active at the start of the experiment, 65 under Ang II and 99 after norepinephrine application. The separation of glycinergic-rhythmic (Figure 5G) and non-fluorescent rhythmic (Figure 5H; neither glycinergic nor GABAergic) neurons revealed that norepinephrine led to a significant increase only in the non-fluorescent group, which are presumably excitatory (Figure 5H; RM one-way ANOVA, Bonferroni adjusted *p*-value: *p* = 0.008). In total 49 non-fluorescent neurons were rhythmically active under control condition, 52 under Ang II and 77 under norepinephrine and in the glycinergic group 13, 13 and 22, respectively. This data is compatible with the notion that Ang II induces only an increase in the respiratory rate but not in the burst amplitude.

### 3.5. Effect of Ang II on non-rhythmic cells of the preBötC

The quantification above was solely based on the correlation of the peaks of the calcium signals with the peaks of the LFP, thus excluding information of neurons that are either inactive or tonic-active. To also quantify if non-rhythmic neurons show increased activity upon Ang II treatment, the intracellular calcium response of the soma was analyzed. A neuron was categorized as responding with a calcium response to the treatment if the fluorescence increased 2× SD from the control fluorescence. Again, by using COFLOUR mice we could study the effect of Ang II on glycinergic, GABAergic as well as GABA-glycine co-transmitting (GGCN) inhibitory neurons. Ang II elicited a response in 26% of all glycinergic neurons while the co-application of norepinephrine induced a calcium rise in 47% glycinergic neurons (Table 1). While both treatments elicited a response in about half of glycinergic neurons identified as rhythmic neurons (Table 1; Ang II: 45.5% vs. NE: 54.5%, *N* = 10 slices), more than double of non-rhythmic glycinergic neurons responded to norepinephrine as compared to Ang II (Ang II: 20.5% vs. NE: 44.9%). While the number of responding GABAergic interneurons was similar between Ang II and norepinephrine (41.7 vs. 50%), Ang II induced a rise in calcium in only half as many GGCN neurons compared to norepinephrine (23.8 vs. 47.6%). Cells in the COFLUOR line that do not express fluorescent proteins (FP) can be considered as excitatory neurons or glia cells. These cells responded to norepinephrine treatment about threefold compared to Ang II. This difference was evident for both non-rhythmic cells (Table 1; Ang II: 25% vs. NE: 76.4%) and rhythmic cells (Ang II: 23% vs. NE: 74.3%).

**TABLE 1:** Number of neurons, which responded to Ang II or to norepinephrine (NE; in the presence of Ang II).

		Ang II		NE (in Ang II)	
	**Total**	**Responsive**	**Responsive (%)**	**Responsive**	**Responsive (%)**
Glycinergic total	100 (10.0 ± 2.67)	26 (2.6 ± 2.59)	26.0	47 (4.7 ± 2.06)	47.0
Glycinergic inspiratory*	22 (2.2 ± 2.30)	10 (1.1 ± 1.76)	45.5	12 (1.3 ± 1.32)	54.5
Glycinergic non-rhythmic	78 (7.8 ± 2.04)	16 (1.6 ± 1.17)	20.5	35 (3.5 ± 1.58)	44.9
GABAergic total	12 (1.2 ± 1.23)	5 (0.6 ± 0.52)	41.7	6 (0.9 ± 1.46)	50.0
GABAergic inspiratory*	–	–	–	–	–
GABAergic non-rhythmic	12	5	41.7	6	50.0
GGCN total	21 (2.1 ± 1.52)	5 (0.6 ± 0.73)	23.8	10 (1.1 ± 1.45)	47.6
GGCN inspiratory*	–	–	–	–	–
GGCN non-rhythmic	21	5	23.8	10	47.6
No FP expressing total	214 (21.4 ± 9.11)	52 (5.2 ± 3.97)	24.3	162 (16.2 ± 7.48)	75.7
No FP expressing inspiratory*	74 (6.6 ± 6.72)	17 (1.8 ± 2.45)	23.0	55 (4.8 ± 5.36)	74.3
No FP expressing non-rhythmic	140 (14 ± 6.18)	35 (3.5 ± 2.88)	25.0	107 (10.7 ± 1.48)	76.4

A neuron was categorized as responding to the treatment if the intracellular calcium concentration increased 2 × SD from the mean under the control condition. Glycinergic, GABAergic, and GABA-glycine co-transmitting (GGCN) inhibitory neurons were identified using COFLOUR mice. Rhythmic neurons were identified *via* the correlation of the calcium responses to the LFP rhythmic bursts. Values in brackets are MEAN ± SD, *N* = 10 slices. *Including all cells that showed inspiratory calcium signals (some of them only after application of NE). FP: fluoresent protein.

### 3.6. Ang II does not induce astrocytic calcium signaling

**FIGURE 6:**
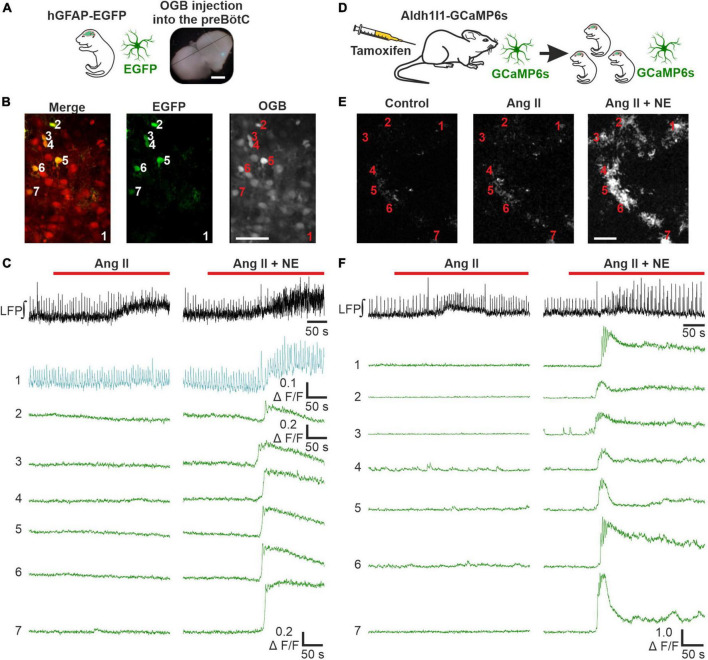
Ang II does not lead to an increase in intracellular calcium in astrocytes. LFP recordings were combined with 2P microscopy in acute rhythmic slices. Two mouse models were used to investigate the effect of Ang II on astrocytes. **(A–C)** Acute slices of mice expressing EGFP in astrocytes were injected with OGB. **(A)** Scale bar 1 mm. **(B)** Exemplary images for EGFP and OGB. **(D–F)** Mice expressing GCaMP6s in astrocytes conditionally, were injected with tamoxifen on two consecutive days postpartum. The pups receive tamoxifen through the mother’s milk, allowing direct calcium-imaging of astrocytes. The numbers in the images **(B,E)** indicate the cells for which the calcium-traces are shown **(C,F)**. **(B,E)** Scale bars 50 μm. **(C,F)** Co-treatment with norepinephrine (NE) served as a positive control after the Ang II application. **(A–C)** *N* = 11 slices, *n* = 70 astrocytes and **(D–F)** *N* = 9 slices, *n* = 160 astrocytes.

To discriminate between effects of Ang II on neurons and astrocytes, we next investigated if Ang II has any effect on astrocytic calcium signaling. In a first set of experiments acute respiratory rhythmic slices were prepared from mice expressing EGFP in astrocytes (hGFAP-EGFP) and OGB was used as calcium indicator (Figures 6A, B). Norepinephrine was used as a positive control in these experiments and elicited a clear calcium response in astrocytes (Figure 6C), as reported previously (Schnell et al., 2016). Interestingly, a comparable rise in intracellular calcium was never seen with Ang II application alone (Figure 6C). Out of 163 astrocytes treated with Ang II, only 7 showed a small change in the calcium response, which was however over the threshold of 2 × SD of the baseline (i.e., astrocytes which responded) (Supplementary Figure 1). We suspected these effects to be rather unspecific, potentially due to activity changes in nearby neurons which overlap with the analyzed astrocytes or due to small drift of the tissue during imaging. To exclude neuronal contamination of the “astrocytic” calcium signal, we used another mouse line expressing GCaMP6s in astrocytes (Figure 6D), allowing exclusive calcium imaging of astrocytes (Figure 6E). Norepinephrine again elicited a clear calcium response in all 72 identified astrocytes, while Ang II did not induce any astrocytic calcium signaling, as can be seen in the example traces (Figure 6F).

As a side note, this mouse line allowed us to readdress the question whether there are respiratory rhythmic astrocytes in the preBötC (Schnell et al., 2011; Okada et al., 2012). None of the 72 astrocytes showed calcium signals that were constantly coupled to the peaks of the LFP as described previously (Okada et al., 2012). We could however identify calcium events from a few astrocytes which overlapped with individual LFP bursts. These cells are depicted in a Supplementary Figure 2.

## 4. Discussion

While angiotensin II (Ang II) has been widely studied for its effect on the cardiovascular system for almost a century, little is known regarding the modulation of respiration. To our knowledge, this study describes for the first time the effect of Ang II on the preBötzinger complex (preBötC), the key site essential for the generation of breathing in the brainstem. It is evident that Ang II signaling *via* the AT1R increases respiratory rhythmic neuronal activity in the preBötC. This effect appears to be mediated by neurons, rather than through astroglia, as Ang II does not induce astrocytic calcium signaling.

We show that Ang II also increases phrenic nerve rate in the WHBP, indicating a higher central respiratory drive which concurs with previous findings showing that peripheral infusion of Ang II in dogs stimulates respiration (Ohtake et al., 1993; Ohtake and Jennings, 1993). While Ang II stimulates the afferent activity of the carotid body (CB), it has been suggested that the CB mediates only in part the effect of Ang II on respiration (Potter and McCloskey, 1979), suggesting a potential involvement of respiratory control regions in the brainstem. Indeed, we found that Ang II increases rhythmic activity in the preBötC. This finding corroborates another study showing enhanced non-cardiac rhythmic RVLM presympathetic neuron activity in hypertensive rats treated with Ang II and a high-salt diet (Toney et al., 2010). This is generally in concurrence with other reports showing that Ang II activates (Hirooka et al., 1996) and increases the firing rate of neurons in the RVLM (Chan et al., 1991; Hirooka et al., 1996; Matsuura et al., 2002; Dampney et al., 2007). Furthermore, Ang II-immunoreactive structures have been found in respiratory brainstem regions, such as the Bötzinger complex in the cat (Aguirre et al., 1989). However, our findings contradict a study in which Ang II was microinjected into the RVLM in anesthetized rabbits without affecting phrenic nerve activity (Li et al., 1992). However, this lack of effect of Ang II on respiration might be explained by a specific activation on a subset of neurons by Ang II in discrete brain areas (Aguirre et al., 1989; Li et al., 1992; Dampney, 1994; Hirooka et al., 1996). Therefore, depending on the exact location of the microinjection into the RVLM, Ang II might simply not have reached the regions which are activated by Ang II and control respiration.

There is accumulating evidence that AT2R and MasR activation tend to oppose the actions of those of AT1R in the brain, i.e., vasodilation (von Bohlen und Halbach and Albrecht, 2006; Sumners et al., 2015; Cosarderelioglu et al., 2020). Interestingly, the localization of AT1R differs from that of AT2R in regions involved in cardiovascular control and both receptors are primarily not expressed on the same neuron (Sumners et al., 2020). Since we did not observe any alteration in respiratory rate activating AT2R in the preBötC, at least not with the concentration used, we speculate that AT2R might not be expressed on respiratory neurons. We have also tested the effect of Ang-(1–7), which can be generated from Ang II by the endogenous ACE2 enzymes. Since we did not observe any increase in the respiratory rate by Ang-(1–7), we can exclude that the Ang II induced increase of the respiratory drive is mediated *via* activation of Mas-receptors.

### 4.1. Type of neurons involved in Ang II response

In the RVLM, AT1R expression was reported for glutamatergic and GABAergic neurons (Hu et al., 2002), and it is known that activation of AT1R has a depolarizing effect of neurons (Latchford and Ferguson, 2005). In line with this data, preBötC neurons show rises of the baseline calcium signal in response to Ang II application. In addition to glutamatergic and GABAergic neurons, an increase of the calcium signal was found in glycinergic neurons. We found that Ang II modulated the baseline activity of a higher proportion of GABAergic than glycinergic interneurons (42 vs. 26%). There were also GABA-glycine co-transmitting neurons (GGCN) activated by Ang II. The share of Ang II responding GGCN was similar to the share of glycine inhibitory neurons being modulated by Ang II. Interestingly, when separating glycinergic neurons into two groups, those with inspiratory activity and those without, the share of glycinergic neurons, in which Ang II mediated an increase of baseline calcium concentration was higher in the group of inspiratory glycinergic neurons compared to the non-rhythmic cells (46 vs. 21%). Whether, this network effect depends on a different AT1R distribution among different types of neurons is not clear at present. In general, our data support the notion that Ang II substantially modulates the activity of inhibitory neurons (Yao et al., 2008; Legat et al., 2019; Singh et al., 2021). With respect to the observed effect of Ang II on the respiratory rhythm, we assume that an increase in the number and/or firing rate of tonically active excitatory neurons is involved in the increase of the overall drive of the network (Kam et al., 2013), while a temporal well-coordinated phasic inhibition of excitatory neurons by rhythmic glycinergic neurons might increase the “respiratory” burst-frequency by, e.g., shortening the refractory period (Cregg et al., 2017; Baertsch et al., 2018).

### 4.2. Role of astrocytes

Astrocytes have been discussed to be the major source of angiotensinogen, the precursor molecule for Ang II in the brain (Stornetta et al., 1988; Milsted et al., 1990; Cosarderelioglu et al., 2020). In our experiments we did not detect any astrocytic activity in the preBötC induced by Ang II. The existence of AT1R on astrocytes is still controversial, with studies supporting (Fogarty and Matute, 2001; Garrido-Gil et al., 2013) or disagreeing (O’Callaghan et al., 2011; Gonzalez et al., 2012; Sumners et al., 2020) that astrocytes express AT1R. While this might depend on the brain region (Fogarty and Matute, 2001), there is even conflicting literature within the same brain areas analyzed, as reported in a recent study (Sumners et al., 2020). Sumners et al. (2020) showed that AT1R expression is limited to neurons and not astrocytes, analyzing multiple brain areas (PVN, MnPO, NTS, AP, and OVLT). Our own RNAseq data from astrocytes in brainstem, hippocampus and cortex reported only few copies of AT1R mRNA (Schnell et al., 2015).

Ang II was shown to inhibit the uptake of glutamate by astrocytes, leading to increased ambient glutamate levels and subsequent increased neuronal activity (Stern et al., 2016). Therefore, although we did not detect that Ang II modulates astrocytic calcium activity, it might still be of interest to have a closer look into a potential role of astrocytes in the preBötC mediating the actions of Ang II.

It is debated whether astrocytes exhibit activity, e.g., calcium rises, that correlates and is entrained with preBötC neurons respiratory bursts (Schnell et al., 2011; Okada et al., 2012). Taking advantage of our Aldh1l1-GCaMP6s mouse line, allowing unequivocal identification and imaging of astrocytes without possible confounding effects that come with multi-cell bolus loading, we showed that there was little to no overlap of the calcium signals with the local preBötC respiratory network discharges. Only a few astrocytes showed individual calcium signals, which coincided with the LFP. These events were rare however, and appeared to be rather stochastic and spontaneous fluctuating calcium signals (see Supplementary Figure 2).

### 4.3. Additional effect of Norepinephrine

Previous studies have shown that norepinephrine, like other neuromodulators, stimulates the output of the respiratory network activity (Schnell et al., 2016), which we have confirmed in our study as well. Furthermore, we have also confirmed the notion that norepinephrine modulates the activity of the respiratory network by acting, not only on neurons but also on astrocytes in the preBötC (Viemari and Ramirez, 2006; Schnell et al., 2016). Interestingly, norepinephrine application led to a recruitment of additional rhythmic neurons, which was not the case for Ang II. We speculate that astrocytes might be involved in mediating this effect.

### 4.4. Pathophysiological implications

What are possible implications of this study? Consequences of increased central Ang II beyond the cardiovascular system are not yet well investigated and understood. However, especially regarding respiratory effects, this could be of interest because animal models indicate an involvement of RAS in the pathogenesis of acute respiratory distress syndrome (ARDS). Mouse models of ARDS show increased Ang II levels (Imai et al., 2005; Wösten-van Asperen et al., 2011; Zou et al., 2014) as well as lung injured mice treated with the recombinant SARS-CoV surface-Spike protein (Kuba et al., 2005). Although it is still under debate, whether Ang II levels are regularly elevated in ARDS and COVID-19 patients (Liu et al., 2020; Wu et al., 2020; Files et al., 2021; Ozkan et al., 2021; Rieder et al., 2021; van Lier et al., 2021), our data suggest that Ang II mediated effects on the preBötC core respiratory network might be a mechanism involved in an increased respiratory drive in these patients, which may lead to patient self-inflicted lung injury (Hülsmann et al., 2020; Camporota et al., 2022).

In conclusion, we showed that Ang II signaling *via* the AT1R increases rhythmic drive in the preBötC, involving both excitatory and inhibitory neurons but not astrocytes. Despite our novel findings, further experimental investigations are required to define more precisely the subcellular actions of Ang II on the neuronal network in the preBötC.

## Data availability statement

The raw data supporting the conclusions of this article will be made available by the authors, without undue reservation.

## Ethics statement

The animal study was reviewed and approved by the LAVES.

## Author contributions

SH and CT designed the experiments. CT and AMB performed the experiments. CT analyzed the data, prepared the figures, and wrote the main manuscript. CT, SH, AH, and BV interpreted and discussed the data, edited, and revised the manuscript. SH supervised the project. All authors have read and approved the final version of this manuscript and agreed to be accountable for all aspects of the work in ensuring that questions related to the accuracy or integrity of any part of the work are appropriately investigated and resolved.
