# Supplementary material for: Angiotensin II increases respiratory rhythmic activity in the preBötzinger complex without inducing astroglial calcium signaling
Source: Front Cell Neurosci. 2023 Feb 2;17:1111263. doi: 10.3389/fncel.2023.1111263 (PMC9932970; doi:10.3389/fncel.2023.1111263)
Supplement: Supplementary file 1 [file Data_Sheet_1.PDF]

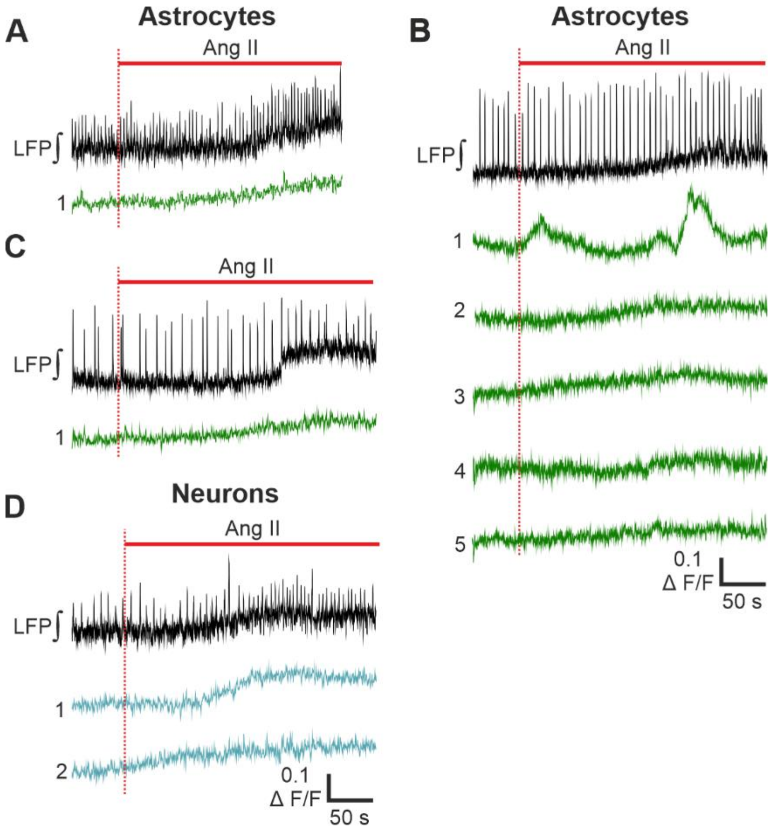

Supplementary Figure 1: Potential astrocytic calcium signals to Ang II in hGFAP-EGFP mice.

(A, B & C) Traces of LFP (black) and calcium recordings (green) of astrocytes from hGFAP-EGFP mice which showed a fluorescent change larger than 2 x SD from the baseline after Ang II application. **(D)** Example traces of calcium recordings from neurons.
