# Supplementary material for: Angiotensin II increases respiratory rhythmic activity in the preBötzinger complex without inducing astroglial calcium signaling
Source: Front Cell Neurosci. 2023 Feb 2;17:1111263. doi: 10.3389/fncel.2023.1111263 (PMC9932970; doi:10.3389/fncel.2023.1111263)
Supplement: Supplementary file 2 [file Data_Sheet_2.PDF]

Tacke et al., Supplemental Figure 2

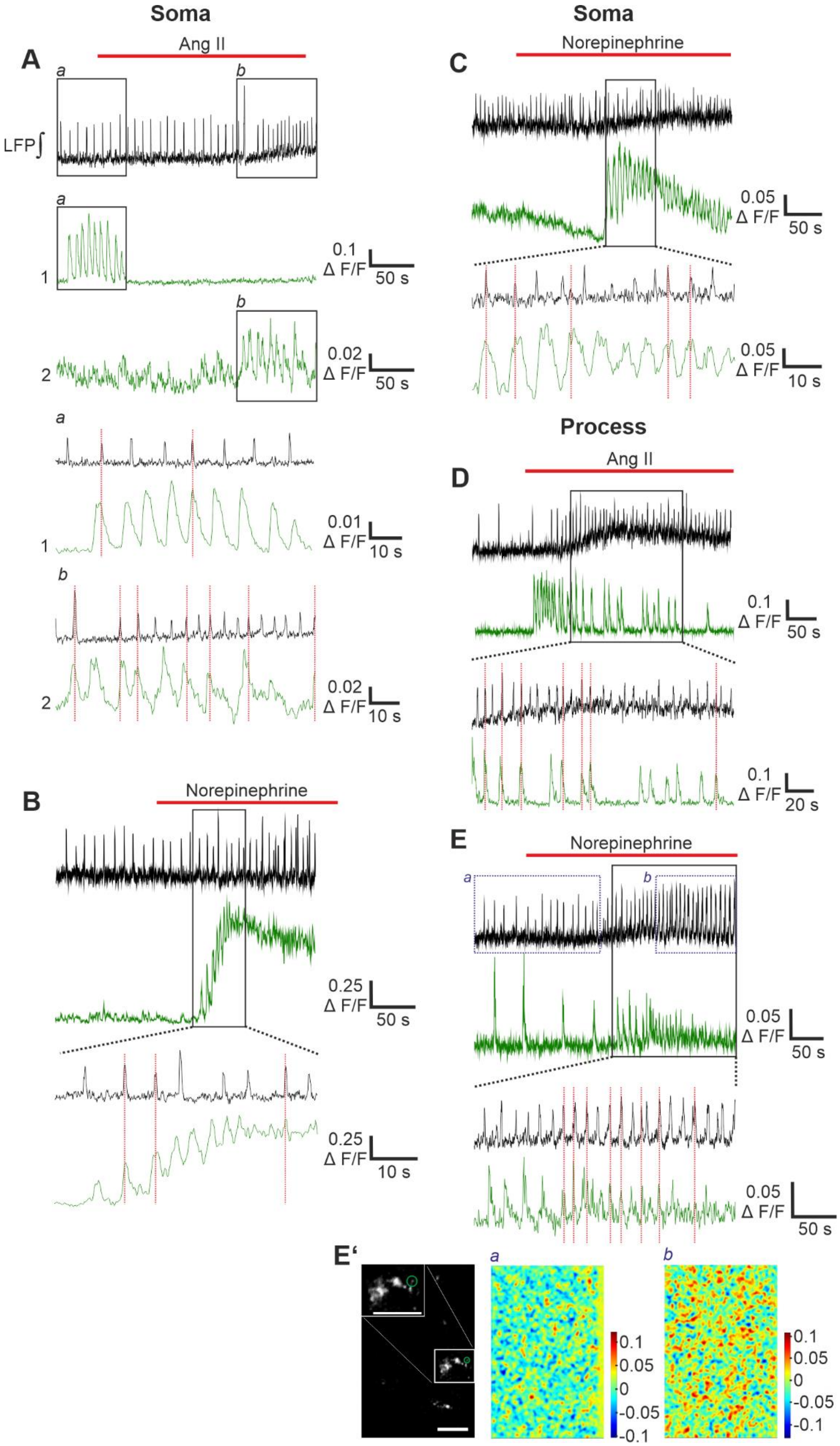

Supplementary Figure 2: Correlation of astrocytic activity with LFP bursts.
Traces of calcium recordings showing partial overlap with LFP bursts (marked with red dotted lines). The entire length of the recordings is shown on top and a selected time frame below, respectively. **(A-C)** Measurement of the soma (including processes) and **(D & E)** only processes of astrocytes. **(E')** Image of astrocyte with the analyzed area (green circle marks process) from which the calcium trace is shown in **(E)**. Scale bars 50 µm. The cross-correlation images of the LFP and the calcium signals were generated from the entire image for two time periods (marked with blue dotted boxes in LFP trace **(E)** labeled *a* and *b*).
